# Moderate, Little, or No Improvements in Neurobehavioral Symptoms among Individuals with Long COVID: A 34-Country Retrospective Study

**DOI:** 10.3390/ijerph191912593

**Published:** 2022-10-02

**Authors:** Daniela Ramos-Usuga, Paul B. Perrin, Yelena Bogdanova, Laiene Olabarrieta-Landa, Elisabet Alzueta, Fiona C. Baker, Stella Iacovides, Mar Cortes, Juan Carlos Arango-Lasprilla

**Affiliations:** 1 Biomedical Research Doctorate Program, University of the Basque Country, Barrio Sarriena, s/n, 48940 Leioa, Spain; 2 Department of Psychology, School of Data Science, University of Virginia, 400 Brandon Ave., #177, Charlottesville, VA 22903, USA; 3 Physical Medicine & Rehabilitation, VA Boston Healthcare System, 150 South Huntington Avenue, Boston, MA 02130, USA; 4 Department of Psychiatry, Boston University School of Medicine, 72 E Concord St, Boston, MA 02118, USA; 5 Health Sciences Department, Public University of Navarre (UPNA), Cataluña, s/n, 31006 Pamplona, Spain; 6 Instituto de Investigación Sanitaria de Navarra (IdiSNA), 31008 Pamplona, Spain; 7 Center for Health Sciences, SRI International, 333 Ravenswood Ave, Menlo Park, CA 94025, USA; 8 School of Physiology, Brain Function Research Group, Faculty of Health Sciences, University of the Witwatersrand, Johannesburg 2000, South Africa; 9 Department of Rehabilitation and Human Performance, Icahn School of Medicine at Mount Sinai, 1 Gustave L. Levy Pl, New York, NY 10029, USA; 10 Departments of Psychology and Physical Medicine and Rehabilitation, Virginia Commonwealth University, 907 Floyd Ave, Richmond, VA 23284, USA

**Keywords:** COVID-19, SARS-CoV-2, Long COVID, neurobehavioral symptoms, risk factors

## Abstract

(1) Background: Some people with COVID-19 develop a series of symptoms that last for several months after infection, known as Long COVID. Although these symptoms interfere with people’s daily functioning and quality of life, few studies have focused on neurobehavioral symptoms and the risk factors associated with their development; (2) Methods: 1001 adults from 34 countries who had previously tested positive for COVID-19 completed the Neurobehavioral Symptom Inventory reporting the symptoms before their COVID-19 diagnosis, during the COVID-19 infection, and currently; (3) Results: Participants reported large-sized increases before vs. during COVID-19 in all domains. Participants reported a medium-sized improvement (during COVID-19 vs. now) in somatic symptoms, a small-sized improvement in affective symptoms, and very minor/no improvement in cognitive symptoms. The risk factors for increased neurobehavioral symptoms were: being female/trans, unemployed, younger age, low education, having another chronic health condition, greater COVID-19 severity, greater number of days since the COVID-19 diagnosis, not having received oxygen therapy, and having been hospitalized. Additionally, participants from North America, Europe, and Central Asia reported higher levels of symptoms across all domains relative to Latin America and Sub-Saharan Africa; (4) Conclusions: The results highlight the importance of evaluating and treating neurobehavioral symptoms after COVID-19, especially targeting the higher-risk groups identified. General rehabilitation strategies and evidence-based cognitive rehabilitation are needed in both the acute and Long COVID phases.

## 1. Introduction

The “Persistent post-COVID syndrome” or “Long COVID” occurs in individuals with a history of probable or confirmed Severe Acute Respiratory Syndrome Coronavirus 2 (SARS CoV-2) infection, usually 3 months from the onset of COVID-19 with symptoms and that last for at least 2 months and cannot be explained by an alternative diagnosis [1]. While these long-term symptoms are more common in individuals with severe COVID-19 presentations, they can also appear in people who had mild infections and had not required hospitalization [2]. Since COVID-19 can affect many organs and bodily systems, the nature of its sequelae is highly variable, including respiratory, cardiovascular, dermatological, muscular, neurological, and psychiatric problems, among others. The most prevalent symptoms reported in the literature include fatigue [2,3,4,5], myalgia [3,4,5], headaches [2,4,5], dyspnea [2,4], cognitive problems [2,4,5], anxiety, and depression [4].

In terms of neurological symptoms, many studies have examined how SARS-CoV-2 affects the central nervous system (CNS). The virus can cause mild disorders such as headache, dizziness, anosmia, or dysgeusia [6,7], but also more serious conditions such as loss of consciousness for prolonged periods of time, ischemic cerebrovascular disease, encephalopathy, acute necrotizing hemorrhagic encephalopathy, encephalitis, meningitis, epileptic seizures, and demyelinating diseases such as Guillain Barré syndrome [6,7,8,9]. According to a recent study [10] the prevalence of these neurological consequences of COVID-19 is 35–85% of individuals. 

Although the mechanisms by which the virus attacks the brain are still a source of debate, three theories have found scientific support: (a) direct virus infection to astrocytes [11], (b) overreaction of the immune system (cytokine storm; [12]), and (c) blockage of blood flow in the brain causing hypoxia [13].

Similar to what typically occurs when any of these three mechanisms are activated, SARS-CoV-2 mediated neurological damage appears to cause short- and long-term sequelae that affect both cognitive functioning and mental health [14]. In fact, there is evidence of the relationship between other viral respiratory infections, such as severe acute respiratory syndrome (SARS), and the development of long-term cognitive and psychiatric disorders [15], with a prevalence of 27–41% during the acute illness [15]. Some individuals infected with SARS-CoV-2 report a variety of persistent symptoms such as mental fatigue, concentration problems, recurrent confusion, forgetfulness, slow thinking, and even disorientation; this clinical picture, known as colloquially as “mental fog,” limits daily functioning and as a result can impact mental health, which in turn can aggravate cognitive symptoms [4]. Thus, some authors suggest that not only do inflammatory components play a role in the genesis of neuropsychological sequelae, but that they may also be exacerbated by psychiatric symptoms such as anxiety [16].

Despite the implications of these symptoms, few studies have been carried out on the cognitive and emotional sequelae in individuals with COVID-19. For example, Miskowiak et al. [17] evaluated the cognitive functioning of 29 individuals 3–4 months after hospital discharge for COVID-19 through the Trail Making Test (TMT) and the Screen for Cognitive Impairment in Psychiatry (SCIP). The results indicated that between 59% and 65% of the sample presented with clinically significant cognitive impairment, with verbal learning and executive functions being the most affected domains. Similarly, Zhou et al. [18] found, through a neuropsychological evaluation, alterations mainly in sustained attention in a group of 29 individuals who had recovered from the virus. Other research groups have explored the presence of cognitive and emotional problems through self-report using online surveys. Using author-customized online questions, Orrú et al. [5] assessed the presence of persistent symptoms after COVID-19 and found that cognitive impairment and loss of concentration were commonly reported. In a study by Frontera et al. [19], people with Long COVID mainly reported anxiety, mental confusion, difficulty concentrating, and forgetfulness; the authors also reported that young Hispanic women were one of the most vulnerable groups to developing persistent symptoms.

Although these studies provide highly relevant information on the possible cognitive and emotional sequelae of individuals who have had COVID-19, more research is needed to better understand the neurobehavioral symptoms of COVID-19 infection and Long COVID. The current literature is lacking in studies with larger sample sizes, from diverse global regions, using standardized/validated instruments, and also lacking in studies exploring other relevant aspects such as risk factors for the development of sequelae and variables that intensify symptoms. To address these limitations, in this study we used the Neurobehavioral Symptom Inventory (NSI), which is the most widely used questionnaire to assess neurobehavioral symptoms in clinical and non-clinical populations [20]. The NSI has been translated and validated into different languages, including Spanish [21], and has good psychometric properties, such as high internal consistency and construct validity [21,22]. Since it is a short self-assessment, it can be included in an online survey, thus reaching a larger sample of participants from different regions of the world.

## 2. Materials and Methods

### 2.1. Participants

To participate in this retrospective study, the inclusion criteria were: (a) age 18 years or older, and (b) self-report of having tested positive for COVID-19 through a viral and/or antigen test. A total of 1049 participants completed the survey. Of those, 23 participants were removed who did not respond to the item asking if they had tested positive for COVID-19, 8 who did not report a diagnosis year, 10 who reported a diagnosis date before 1 March 2020, and 7 who reported a diagnosis date after the date they took the survey or on a date that had not occurred yet. Therefore, the final sample included 1001 participants.

### 2.2. Measures

Through an online survey, the following information was collected: (a) demographic information (gender, age, romantic relationship status, educational background, work status, and country of residence); (b) pre-existing chronic health condition status; (c) COVID-19 infection characteristics and treatment (date of diagnosis, severity of symptoms, and COVID-19 details of medical intervention).

The NSI [23] was used to evaluate persistent symptoms. It is a self-report questionnaire used to assess cognitive, affective, and somatic neurobehavioral symptoms in people with post-concussion syndrome. It consists of 22 items that are scored on a 5-point Likert scale according to symptom severity (0 = None; 1 = Mild; 2 = Moderate; 3 = Severe; 4 = Very Severe). In the current survey, participants were asked to report the presence of each symptom at three different time-points: before COVID-19 diagnosis, during the COVID-19 infection, and now (when completing the survey). The English and previously validated Spanish version of the NSI were used [21], depending on the global region where participants lived.

### 2.3. Procedure

A team of researchers with expertise in COVID-19 from Spain and the United States of America developed the survey, originally in English and then translated into Spanish. The survey was hosted on Qualtrics and, once the study was approved by the Ethics Committee of the Public University of Navarra, it was distributed through (a) professional mailing lists and collaborators’ contact networks; (b) Facebook groups and advertisements; and (c) a database of individuals with COVID-19 from one of the collaborating centers (Icahn School of Medicine at Mount Sinai in New York City, NY, USA). Data collection took place from 9 March to 7 June 2021. Individuals were invited to participate if they had previously tested positive for COVID-19.

Information about the study was included in the first page of the survey, the social media advertisements, and the recruitment email. This information emphasized that participation was voluntary, data would be anonymous, and there was no financial compensation for participation. To proceed to the survey, informed consent was provided by an affirmative answer to the question “Do you want to participate in this study?” The study was conducted in compliance with the declaration of Helsinki.

### 2.4. Data Analyses

All descriptive statistics and analyses were conducted using IBM SPSS Statistics 27. In order to determine whether there were differences in neurobehavioral symptoms by domain (subscale) and by symptom (item) as recalled before participants’ COVID-19 diagnosis, during the COVID-19 infection, and now (when completing the survey), a series of paired-samples t-tests was conducted. In each analysis, the independent variable was time (before COVID-19 diagnosis, during the COVID-19 infection, and now), and the dependent variable was the NSI subscale score or item. For each analysis, a Cohen’s d effect size was calculated taking into account the longitudinal correlation between subtotal scores or items. Because of the large sample size, Cohen’s d cutoffs and descriptors of 0.2 (small), 0.5 (large), and 0.8 (large) were used rather than *p*-values.

In order to investigate demographic characteristics, presence of a chronic health condition, COVID-19 medical intervention characteristics, COVID-19 symptom severity, and days since COVID-19 diagnosis as predictors of current neurobehavioral symptoms, three hierarchical stepwise linear regressions were computed with neurobehavioral symptom domain (somatic, cognitive, or affective) as the outcome variable. For reference, a bivariate correlation matrix was created between these sets of relationships as well. In these regressions, demographic characteristics (man vs. woman or non-binary/trans, age, education level [with continuous coding as specified in Table 1], employed vs. unemployed [with full-time, part-time, and student coded as “employed” and all other categories coded as “unemployed”], and romantically partnered vs. not partnered) and whether participants reported another chronic health condition were included as Step 1 variables. Step 2 included COVID-19 intervention characteristics (hospitalization, oxygen therapy, Intensive Care Unit (ICU) stay, noninvasive ventilation, invasive ventilation, and induced coma), participants’ self-reported level of COVID-19 symptom severity while positive, and number of days since the COVID-19 diagnosis. 

Then, neurobehavioral symptom category scores were compared by global region using analyses of covariance (ANCOVAs). Demographics included in Step 1 of the previous regressions were included as covariates. Participants from South Asia, as well as East Asia and the Pacific were excluded from the ANCOVAs because the small group size precluded meaningful comparisons (*n* = 4).

## 3. Results

### 3.1. Sociodemographic Characteristics

A total of 1001 participants from 34 different countries (see Supplementary Material S1) completed the survey. Most of the participants were women (*n* = 782; 78%) with a mean age of 43.5 years (SD = 11.9), and with university studies (*n* = 713; 71%). More details of the sociodemographic information can be found in Table 1.

### 3.2. Changes in Neurobehavioral Symptoms over Time

The results of the paired-samples *t*-tests comparing neurobehavioral symptoms before the COVID-19 diagnosis, during the COVID-19 infection, and now (when completing the survey) are presented in Table 2. At the symptom domain level, participants reported large-sized increases during COVID-19 vs. before infection in all domains (somatic, cognitive, and affective). Participants reported a medium-sized improvement (during COVID-19 vs. now) in somatic symptoms and a small-sized improvement in affective symptoms, but the improvement in cognitive symptoms was very minor and did not reach a small-sized effect.

Within the somatic symptom domain, the largest symptom increase during vs. before COVID-19 occurred with changed in taste/smell, appetite, and dizziness. Changes in taste/smell and appetite-related symptoms showed large-sized improvements (now vs. during COVID-19). Within the cognitive symptom domain, the large-sized symptom increases during vs. before COVID-19 occurred with concentration and slowed thinking. All four cognitive symptoms showed extremely low levels of improvement (now vs. during COVID-19) with only concentration symptoms just surpassing the threshold for a small-sized effect and forgetfulness showing no improvement. Within the affective symptom domain, large-sized symptom increases during vs. before COVID-19 occurred with fatigue and headaches. These two symptoms also showed medium-sized improvements (now vs. during COVID-19), although improvements in difficulty falling or staying asleep, irritability, and frustration tolerance failed to reach a small-sized effect.

In the first hierarchical linear regression predicting current somatic symptom severity, Step 1 was statistically significant, *F*(6, 994) = 16.56, *R^2^* = 0.091, *p* < 0.001 (Table 3 with standardized β weights). Greater somatic symptom severity was significantly and uniquely associated with female or trans vs. male gender, lower education level, being unemployed, and having another chronic health condition. With the addition of COVID-19 intervention characteristics, COVID-19 symptom severity, and number of days since the COVID-19 diagnosis as a predictor in Step 2, the overall model was still statistically significant, *F*(14, 986) = 18.80, *R^2^* = 0.211, *p* < 0.001. Within this step, all previously significant predictors were still statistically significant and associations in the same directions, with the addition of greater age now significantly predicting lower somatic symptom severity. Greater somatic symptom severity was uniquely associated with higher COVID-19 severity while positive and greater number of days since participants’ COVID-19 diagnosis.

In the second hierarchical linear regression predicting current cognitive symptom severity, Step 1 was statistically significant, *F*(6, 994) = 11.62, *R^2^* = 0.066, *p* < 0.001 (Table 3). Greater cognitive symptom severity was significantly and uniquely associated with female or trans vs. male gender, being unemployed, and having another chronic health condition. With the addition of the Step 2 predictors, the overall model was still statistically significant, *F*(14, 986) = 16.98, *R^2^* = 0.194, *p* < 0.001. Within this step, all previously significant predictors were still statistically significant and associations in the same directions, with the addition of greater age now significantly predicting lower cognitive symptom severity. Greater cognitive symptom severity was uniquely associated with having been hospitalized, higher COVID-19 severity while positive, and greater number of days since participants’ COVID-19 diagnosis. Conversely, lower cognitive symptom severity was associated with having received oxygen therapy. 

In the third hierarchical linear regression predicting current affective symptom severity, Step 1 was statistically significant, *F*(6, 994) = 19.16, *R^2^* = 0.104, *p* < 0.001 (Table 3). Greater affective symptom severity was significantly and uniquely associated with female or trans vs. male gender, younger age, lower education, being unemployed, and having another chronic health condition. With the addition of the Step 2 predictors, the overall model was still statistically significant, *F*(14, 986) = 21.23, *R^2^* = 0.232, *p* < 0.001. Within this step, all previously significant predictors were still statistically significant and associations in the same directions. Greater affective symptom severity was uniquely associated with higher COVID-19 severity while positive and greater number of days since participants’ COVID-19 diagnosis.

Correlations between neurobehavioral symptoms and predictors can be found in Supplementary Material S2.

### 3.3. Differences in COVID-19 Neurobehavioral Symptoms by Global Region

**Figure 1:**
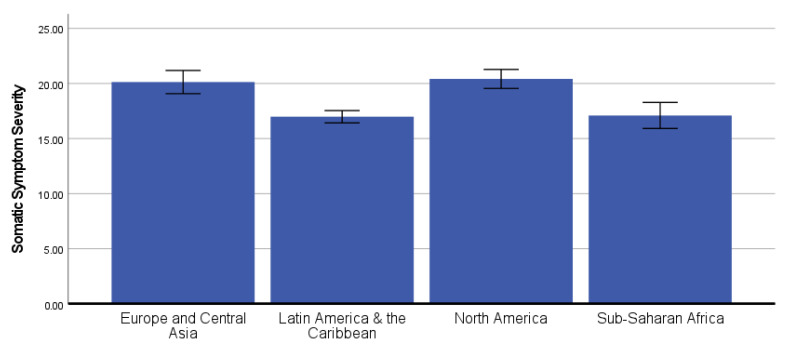
Covariate-adjusted somatic symptom severity scores (mean with 95% confidence interval) by global region. **Note**: A graphic that shows the differences in somatic symptoms by Global Region. The participants from Europe and Central Asia and North America reported significantly greater somatic symptom severity than participants from Latin America and the Caribbean and Sub-Saharan Africa.

In the ANCOVA predicting current somatic symptom severity, there was a statistically significant effect of global region, *F*(3, 987) = 19.67, *p* < 0.001, partial-eta^2^ = 0.056 (Figure 1). Bonferroni-corrected post hoc pairwise comparisons showed that participants from Europe and Central Asia (covariate-adjusted *M* = 20.13, *SE* = 0.54) and North America (covariate-adjusted M = 20.42, SE = 0.44) reported significantly greater somatic symptom severity than participants from Latin America and the Caribbean (covariate-adjusted *M* = 16.99, *SE* = 0.29) and Sub-Saharan Africa (covariate-adjusted *M* = 17.10, *SE* = 0.60), all *p* < 0.001.

**Figure 2:**
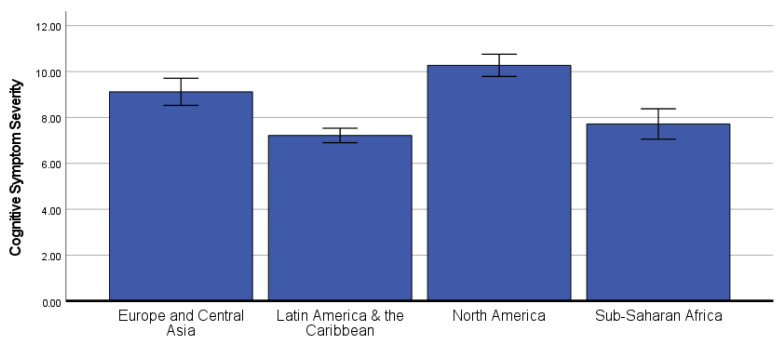
Covariate-adjusted cognitive symptom severity scores (mean with 95% confidence interval) by global region. **Note**: A graphic that shows the differences in cognitive symptoms by Global Region. The participants from North America reported the greatest cognitive symptoms. Additionally, participants from Europe and Central Asia reported significantly greater cognitive symptom severity than participants from Latin America and the Caribbean and Sub-Saharan Africa.

In the ANCOVA predicting current cognitive symptom severity, there was a statistically significant effect of global region, *F*(3, 987) = 39.31, *p* < 0.001, partial-eta^2^ = 0.107 (Figure 2). Bonferroni-corrected post hoc pairwise comparisons showed that participants from Europe and Central Asia (covariate-adjusted *M* = 9.12, *SE* = 0.30) reported significantly greater cognitive symptom severity than participants from Latin America and the Caribbean (covariate-adjusted *M* = 7.22, *SE* = 0.16, *p* < 0.001) and Sub-Saharan Africa (covariate-adjusted *M* = 7.72, *SE* = 0.34, *p* = 0.013), but lower severity than participants from North America (covariate-adjusted *M* = 10.28, *SE* = 0.25, *p* = 0.019). Participants from North America also reported greater cognitive symptom severity than those from Latin America and the Caribbean (*p* < 0.001) and Sub-Saharan Africa (*p* < 0.001).

**Figure 3:**
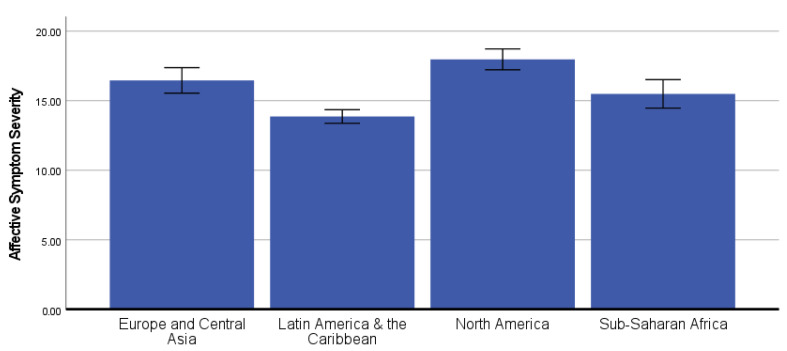
Covariate-adjusted affective symptom severity scores (mean with 95% confidence interval) by global region. **Note**: A graphic that shows the differences in affective symptoms by Global Region. The participants from Latin America and the Caribbean reported significantly lower affective symptom severity than participants from Europe and Central Asia, North America, and Sub-Saharan Africa. Participants from Sub-Saharan Africa also reported lower affective symptom severity than those from North America.

In the ANCOVA predicting current affective symptom severity, there was a statistically significant effect of global region, *F*(3, 987) = 29.26, *p* < 0.001, partial-eta^2^ = 0.082 (Figure 3). Bonferroni-corrected post hoc pairwise comparisons showed that participants from Latin America and the Caribbean (covariate-adjusted *M* = 13.87, *SE* = 0.25) reported significantly lower affective symptom severity than participants from Europe and Central Asia (covariate-adjusted *M* = 16.46, *SE* = 0.47, *p* < 0.001), North America (covariate-adjusted *M* = 17.98, *SE* = 0.38, *p* < 0.001), and Sub-Saharan Africa (covariate-adjusted *M* = 15.50, *SE* = 0.52, *p* = 0.030). Participants from Sub-Saharan Africa also reported lower affective symptom severity than those from North America (*p* < 0.001).

## 4. Discussion

This retrospective study (a) compared the presence of somatic, cognitive, and emotional neurobehavioral symptoms in a large international sample of individuals before their COVID-19 diagnosis, during the COVID-19 infection, and currently, and (b) examined risk factors for increased Long COVID neurobehavioral symptoms including demographic variables, COVID-19 infection severity, and intervention characteristics. Participants showed large-sized symptom increases in all three neurobehavioral domains (somatic, cognitive, and affective) during their COVID-19 infection, consistent with commonly reported COVID-19 symptoms [2,3,4,5]. However, there were marked differences in the patterns of post COVID-19 recovery as a function of symptom domain. Specifically, changes in smell, appetite, and dizziness (somatic symptoms) showed large increases during participants’ COVID-19 infection, with only smell showing a large improvement post COVID-19. Within the affective domain, fatigue and headaches showed large increases during COVID-19, but also medium-sized improvements post COVID-19; whereas there were extremely minimal improvements in sleep, irritability, and frustration. Regarding cognitive symptoms, concentration problems and slowed thinking demonstrated the largest increases during COVID-19. However, all four cognitive symptoms showed very low levels of improvement or no improvement (forgetfulness). The distinct long recovery trajectories in these domains may reflect multiple factors, ranging from pre-existing individual health conditions and vulnerabilities, to specific pathway/s of viral attack, or specific brain regions or neural networks affected by the virus. Further research, and more specifically, longitudinal, neuroimaging, and neurobiological studies, may provide further evidence and insight into specific neurobiological mechanism/s of cognitive impairments associated with COVID-19 infection and help identify additional risk and protective factors.

This study identified several risk factors associated with higher symptom severity. First, the sociodemographic characteristics consistently associated with greater severity of symptoms across the three domains were being female, younger, lower education, being unemployed, and having another chronic health condition. These findings are consistent with a study by Frontera et al. [19], in which participants reporting prolonged COVID-19 symptoms were more often younger, female, Hispanic, with a history of a mental health disorder, unemployed, and with financial insecurity. In fact, several studies from different geographic regions have also reported that females have been disproportionately affected by Long COVID as compared to males [3,24,25]. Moreover, other studies have found that females have experienced higher psychosocial distress and neurobehavioral symptoms than males during the COVID-19 pandemic, even those who had not contracted COVID-19 [19,26,27]. According to Kolakowsky-Hayner et al. [28], these differences may be due to genetics or hormonal factors, structural gender inequity at the societal level, or coping strategies. Therefore, women’s susceptibility to reporting greater mental health symptoms may exacerbate neurocognitive symptoms after COVID-19 infection and maintain them over time.

On the other hand, though older adults are at a greater risk for serious complications of COVID-19, several cross-sectional studies in the general population reported age differences in mental health outcomes during the COVID-19 pandemic, with the younger group being the most psychologically impacted by the pandemic [29,30,31]. Older adults have been shown to have better emotional regulation and engage in proactive coping [32,33], as well as better emotional well-being, a more positive outlook, and emotional resilience, despite prolonged stress of the COVID-19 pandemic [34]. This population also has better coping skills and more stable social/family connections than younger adults which may serve as protective factors against psychosocial distress during the pandemic [31]. The current findings provide additional evidence of this hypothesis. 

The current study results showed that participants with lower education levels reported higher somatic and affective symptoms. While there were no reports in the literature directly addressing the association between low educational level and somatic and affective neurobehavioral symptoms in Long COVID, a cross-sectional study from Poland (*n* = 1002) reported that higher education level and male gender were associated with lower psychopathological symptoms in a general sample during the COVID-19 pandemic. The authors also reported that greater knowledge about COVID-19 was associated with lower symptom severity [26]. Another study showed that greater knowledge about COVID-19 was associated with a more optimistic outlook and the use of preventive measures in a general Chinese sample [35]. Both studies reported that greater knowledge was associated with higher education and the male gender in their respective countries. In many societies and cultures across the globe, higher education is often a pre-requisite and a pathway to a higher paying job/occupation, and consequently, may provide better access to medical care, child and elder care, access to information and support systems, shelter, and food security.

Regarding unemployment, our results coincide with that found by Frontera et al. [19], where this variable is associated with persistent symptoms after COVID-19. Some reports indicated that low socio-economic level, escalated during a public health crisis, can have an adverse effect on physical and mental health and has been associated with higher levels of psychosocial distress [36,37]. Additionally, the lack of employment and low economic resources may mean less access to health care in some regions where healthcare is private, limiting one’s ability to obtain medical assistance, assistance to rehabilitation programs, and needed medications, etc. Likewise, being unemployed is a highly stressful situation for many that may be at the root of many of the affective symptoms reported by the participants of the present study, such as anxiety. Future studies should investigate this potential explanation further, comparing the presence of persistent symptoms in people with high and low socioeconomic status, or employed vs. unemployed. 

Having another chronic health condition was also associated with all three neurobehavioral domains. This is consistent with previous research that has found that individuals with previous chronic diseases such as diabetes [38], asthma [39], hypertension [40], cardiovascular diseases [41], and malignancy [42], or human immunodeficiency virus (HIV) [43] have more complications from being infected with SARS-CoV-2 than those without chronic health conditions. This is possibly due to two mechanisms: (a) a deficient immune system, as in the case of HIV or cancer, or (b) some diseases such as diabetes cause an increase in Angiotensin-Converting Enzyme 2 (ACE-2) receptors, proteins to which SARS-CoV-2 binds to infect cells [44]. The results of the present study increase the evidence of the vulnerability of individuals with chronic health conditions.

Some studies have documented a relationship between the presence of persistent symptoms and greater severity of COVID-19, the need for mechanical ventilation, an ICU stay, and a longer hospitalization [24]. For example, Daste et al. [45] found that 3 months after ICU discharge, persons reported clinically relevant physical impairment (shoulder and peripheral nerve injuries), cognitive alterations (in memory, attention, processing speed, and executive function), and emotional problems (anxiety, depression, and posttraumatic stress disorder). Many of these authors have suggested that people with these characteristics have long-term tissue damage that may be associated with persistent symptoms. In the current sample, greater COVID-19 infection severity predicted greater current symptom severity across all three neurobehavioral domains, however, the other variables such as ICU stay, noninvasive or invasive ventilation, and induced coma were not associated with Long COVID symptoms. The inconsistency and variability of reported post-COVID-19 outcomes in the current literature are likely to reflect the heterogeneity of the population samples, assessment measures, clinical presentations, and recovery trajectories of individuals with prolonged COVID-19 symptoms.

The current study results also showed that cross-sectionally across the sample, greater time since the infection was associated with increased neurobehavioral symptoms. This finding may potentially reflect the natural course of post-COVID recovery, where milder symptoms get resolved soon after the infection, and more severe symptoms continue to develop and/or linger for a longer period of time. However, the emerging literature investigating post-COVID recovery and Long COVID symptoms indicates the possibility of different trajectories determined by distinct temporal subtypes or phenotypes [46]. For instance, a study by Davis et al. [46] reported a continuous increase in cognitive symptoms (brain fog, attention, and memory impairment) in the first few months after the infection which is consistent with the current findings of persistent cognitive symptoms showing no improvement over time in the longitudinal analyses. At this point in time, only limited data are available on the temporal recovery trajectory of Long COVID, and future longitudinal studies using large samples may provide further insight and help to identify and evaluate potential phenotypes.

Lower cognitive symptom severity was uniquely predicted by having received oxygen therapy. Though there is limited literature available on oxygen therapy in individuals with COVID-19, it has been suggested that oxygen treatment, and hyperbaric oxygen therapy in particular, may improve oxygenation and reduce tissue inflammation [47]. A systematic review reported that hyperbaric oxygen treatment improved severe COVID-19 symptoms and increased general wellbeing, while correcting hypoxia and elevating O2 saturation, in several small samples of individuals with COVID-19 [48]. To the authors’ knowledge, the current study is the first to report an association between having received oxygen therapy during COVID-19 and lower cognitive symptoms post COVID-19 infection in a large international sample.

Finally, after controlling for demographics, participants from North America, Europe, and Central Asia generally reported the highest levels of symptoms across all domains relative to other global regions. Although it may seem counterintuitive that people from high-income countries present more sequelae than those from low-income countries taking into account that they have greater access to resources such as health, hygiene, food, etc., data from the Institute of Health Metrics and Evaluation [49] indicated that the incidence of mental disorders in the North American, European, and Central Asian regions were much higher than in Latin America and Africa. These alterations, as suggested by Benzakour et al. [16], can exacerbate cognitive problems, which would explain the results obtained in the present study. However, further research is required in this area. 

## 5. Implications

Compared to previous literature, this study has a number of strengths that should be highlighted. Most of the research carried out on COVID-19 or Long COVID is based on case studies or small samples. The current study presents results obtained from a sample of more than 1000 people which facilitates generalization of the results. In addition, the participants came from different countries, allowing comparisons by global regions which is unique in the literature to date. Although there is much research on certain risk factors that promote the development of Long COVID symptomatology, in the present study, novel factors such as low education and the absence of oxygen therapy were identified as important that have not been previously taken into account. In fact, these unique characteristics predictive of Long COVID symptoms underscore risk factors related to healthcare access, including unemployment, education, and services such as oxygen therapy. Nonetheless, the sample was a convenience sample and therefore not representative in terms of age, education, or geographic region, so conclusions must be tempered accordingly. Clinically, these findings have important implications for Long COVID, highlighting the necessity to assess, monitor, and treat somatic, cognitive, and affective neurobehavioral symptoms in the general population during and after COVID-19, especially targeting the most at-risk groups for higher Long COVID symptom severity (younger age, female or trans gender, unemployed, and having chronic health conditions and lower educational level). In addition, periodic evaluations of these patients must be carried out, considering that the symptoms, in many cases, tend to persist over time. 

Moreover, these results open the way to new research that investigates the characteristics of the presentation of symptoms, and taking into account, mainly, the differences between the different regions of the world. Based on our results, it is possible that there are other environmental and cultural factors that are not being taken into account and that may be influencing not only the appearance of symptoms, but also their reporting. In sum, the risk factors identified provide evidence and future directions for both research and clinical developments.

## 6. Limitations

The results of current study must be interpreted in light of several limitations. The inconsistency and variability of reported post-COVID-19 outcomes in the current literature are likely to reflect the heterogeneity of the population samples, assessment measures, clinical presentations, and recovery trajectories of individuals with prolonged COVID-19 symptoms. For instance, first, the survey was distributed online, so the veracity of what was reported by participants cannot be guaranteed versus if objective indices of the constructs had been collected (e.g., neuropsychology test performance, sleep monitoring study, etc.). Second, people with limited or no access to the internet were unable to participate in this study; previous research has suggested that people from low or very low socioeconomic areas may be differentially impacted by the COVID-19 pandemic compared to those in higher socioeconomic areas [50]. Therefore, the current study likely over-sampled participants of higher educational and income backgrounds. Third, as the study was retrospective, participants responded according to their recall of symptoms at various stages in the COVID-19 infection course. Participants with greater psychological distress may have had a more biased, exacerbated recall of their COVID-19 symptoms or premorbid functioning. Fourth, this study was only focused on adults with COVID-19, and future studies should include adolescents and children to increase the field’s knowledge about neurobehavioral symptoms in Long COVID among these groups as well. Fifth, although data were collected from 34 countries, the fact that the survey was only available in English and Spanish likely limited the number of participants from Asia, Africa, or Europe. Sixth, participants with severe and critical symptoms are underrepresented in the cohort since they represent only 11% of the sample. Seventh, given the study’s recruitment approach, the total number of individuals who saw the study invitation is unknown, and differences between those who participated vs. did not participate in the study are similarly unknown. Finally, as vaccine campaigns increase, future studies should follow up with people who have received the vaccine and become infected after vaccination to determine whether neurobehavioral symptomatology is similar or different compared to those who are unvaccinated.

## 7. Conclusions

This study found that COVID-19 might result in long-term neurobehavioral symptomatology, with medium-size improvements in somatic and affective domains, but small-sized or non-existent improvements in the cognitive domain. Evaluation and monitoring of neurobehavioral symptoms after COVID-19 are warranted. Moreover, the results reported here allow clinicians to identify early individuals who are at an increased risk of developing long-term neurobehavioral symptoms, and thus start with early intervention for groups including: younger age, being female or trans, having low education, having another chronic health condition, being unemployed, and higher COVID-19 severity. Finally, global region differences arose, with North America, Europe, and Central Asia participants generally reported the highest levels of symptoms across all domains relative to other global regions. This may reflect social inequalities making the COVID-19 pandemic worse in particular global regions.

## Figures and Tables

**Table 1:** Sociodemographic characteristics of the study cohort (N = 1001).

**Variable**	**Value**	
Age (years), M ^1^, SD ^2^	43.5	12
Gender, N, %		
Man	213	21
Woman	782	78
Non-binary, transgender, or other	6	1
Country region, N, %		
Europe and Central Asia	147	15
Latin America and the Caribbean	516	51
North America	218	22
South/East Asia and the Pacific	4	0.4
Sub-Saharan Africa	116	12
Work status, N, %		
Full-time employed	571	57
Part-time employed	145	14
On leave	63	6
Volunteering	10	1
Student	48	5
Unemployed	56	6
Retired	42	4
Staying at home/homemaker	49	5
Disability	17	2
Highest level of education completed, N, %		
Some primary education (elementary school)	5	0.5
Completed primary education (graduated elementary school)	5	0.5
Some secondary education (high school)	10	1
Completed secondary education (graduated high school)	57	6
Trade/technical/vocational training	90	9
Some undergraduate education (college or university)	121	12
Completed undergraduate education	301	30
Some postgraduate education	145	15
Completed postgraduate education (masters or doctorate)	267	27
Romantic relationship status, N, %		
Partnered	717	72
Single	284	28
Past chronic health condition, N, %		
At least one past chronic health conditions	586	59
No other chronic health condition	415	41
Hospitalized, N, %	111	11
Oxygen Therapy, N, %	82	8
ICU ^3^ Stay, N, %	25	3
Noninvasive Ventilation, N, %	15	2
Invasive Ventilation, N, %	7	0.7
Induced Coma, N, %	5	0.5
Days from COVID-19 diagnosis M, SD	175	118
Severity of the symptoms while positive for COVID-19, N, %		
No symptoms (asymptomatic)	35	4
Some mild symptoms (no need for treatment)	309	31
Moderate symptoms (needed treatment, but no hospitalization)	546	55
Severe symptoms (hospitalization)	84	8
Critical symptoms (intensive care unit)	27	3

^1^ Mean; ^2^ Standard Deviation; ^3^ Intensive care unit.

**Table 2:** Neurobehavioral symptoms before COVID-19 diagnosis, during COVID-19 infection, and now (N = 1001).

Neurobehavioral Symptom	*M*(*SD*) Before	*M*(*SD*) During	*M*(*SD*) Now	*d* before vs. during	*d* before vs. Now	*d* during vs. Now
Somatic	13.33(3.25)	21.74(7.96)	18.20(6.92)	−1.15	−0.76	0.61
Dizzy	1.22(0.51)	2.15(1.09)	1.75(0.92)	−0.87	−0.58	0.40
Balance	1.14(0.43)	1.79(1.03)	1.53(0.82)	−0.66	−0.51	0.31
Coordination	1.16(0.44)	1.76(1.00)	1.56(0.84)	−0.63	−0.51	0.25
Nausea	1.13(0.44)	1.89(1.10)	1.48(0.84)	−0.73	−0.43	0.44
Vision	1.36(0.65)	1.86(1.00)	1.82(0.96)	−0.57	−0.55	0.06
Light Sensitivity	1.29(0.63)	1.80(1.06)	1.60(0.90)	−0.54	−0.40	0.26
Hearing	1.22(0.55)	1.44(0.78)	1.45(0.78)	−0.35	−0.37	−0.02
Noise Sensitivity	1.27(0.58)	1.75(1.02)	1.66(0.92)	−0.54	−0.48	0.12
Numb/Tingling	1.27(0.55)	1.94(1.09)	1.81(0.99)	−0.65	−0.58	0.14
Smell	1.07(0.38)	2.85(1.45)	1.80(1.07)	−1.21	−0.68	0.84
Appetite	1.19(0.52)	2.50(1.20)	1.72(0.95)	−1.05	−0.55	0.67
Cognitive	5.09(1.68)	8.74(4.15)	8.21(3.94)	−0.91	−0.81	0.17
Concentration	1.29(0.56)	2.43(1.22)	2.20(1.15)	−0.95	−0.80	0.22
Forgetfulness	1.37(0.59)	2.22(1.17)	2.23(1.11)	−0.76	−0.78	−0.01
Making Decisions	1.22(0.49)	1.88(1.09)	1.75(0.98)	−0.65	−0.57	0.17
Slowed Thinking	1.21(0.51)	2.20(1.21)	2.03(1.12)	−0.85	−0.75	0.17
Affective	10.52(3.57)	17.38(6.04)	15.33(6.13)	−1.24	−0.86	0.46
Headaches	1.71(0.87)	2.87(1.19)	2.16(1.08)	−0.93	−0.41	0.63
Fatigue	1.36(0.63)	3.35(1.18)	2.64(1.20)	−1.61	−1.05	0.62
Sleep	1.50(0.78)	2.35(1.29)	2.24(1.18)	−0.68	−0.69	0.11
Anxious	1.63(0.78)	2.48(1.19)	2.24(1.09)	−0.77	−0.61	0.26
Depressed	1.43(0.72)	2.23(1.19)	2.04(1.10)	−0.74	−0.60	0.20
Irritability	1.49(0.68)	1.99(1.03)	1.98(1.01)	−0.54	−0.54	0.02
Frustration	1.41(0.64)	2.10(1.16)	2.03(1.10)	−0.66	−0.62	0.09

**Table 3:** Neurobehavioral symptom domain multiple regressions with standardized B-weights and *p*-value (N = 1001).

	Somatic		Cognitive		Affective	
Predictor	β	*p*-Value	β	*p*-Value	β	*p*-Value
Step 1:						
Male Gender	−0.13	<0.001	−0.09	0.004	−0.12	<0.001
Age	0.00	0.896	−0.03	0.364	−0.06	0.040
Education	−0.08	0.013	−0.01	0.859	−0.10	0.002
Employed	−0.16	<0.001	−0.16	<0.001	−0.16	<0.001
Partnered	−0.06	0.072	−0.06	0.071	−0.04	0.178
Other Chronic Condition	0.15	<0.001	0.14	<0.001	0.19	<0.001
Step 2:						
Male Gender	−0.15	<0.001	−0.10	<0.001	−0.14	<0.001
Age	−0.07	0.031	−0.08	0.007	−0.12	<0.001
Education	−0.11	<0.001	−0.04	0.171	−0.13	<0.001
Employed	−0.10	0.001	−0.11	<0.001	−0.10	0.001
Partnered	−0.04	0.155	−0.04	0.162	−0.03	0.313
Other Chronic Condition	0.12	<0.001	0.12	<0.001	0.16	<0.001
Hospitalized	0.08	0.198	0.18	0.003	0.08	0.192
Oxygen Therapy	−0.01	0.898	−0.15	0.006	−0.06	0.282
ICU Stay	−0.02	0.769	−0.05	0.303	−0.09	0.062
Noninvasive Ventilation	0.00	0.924	−0.02	0.748	0.02	0.710
Invasive Ventilation	−0.02	0.707	−0.02	0.744	−0.02	0.788
Induced Coma	0.04	0.422	0.02	0.695	0.07	0.223
COVID−19 Severity	0.24	<0.001	0.27	<0.001	0.29	<0.001
Days Since Diagnosis	0.18	<0.001	0.20	<0.001	0.19	<0.001

## Data Availability

Not applicable.

## Supplementary Materials

The following supporting information can be downloaded at: https://www.mdpi.com/article/10.3390/ijerph191912593/s1, S1: Sample Size by countries; S2: Bivariate correlations (r) between neurobehavioral symptoms and predictors.
