# Supplementary material for: Moderate, Little, or No Improvements in Neurobehavioral Symptoms among Individuals with Long COVID: A 34-Country Retrospective Study
Source: Int J Environ Res Public Health. 2022 Oct 2;19(19):12593. doi: 10.3390/ijerph191912593 (PMC9564784; doi:10.3390/ijerph191912593)
Supplement: Supplementary file 1 [file ijerph-19-12593-s001.zip › ijerph-1863292-supplementary.pdf]

Supplementary material S1.Sample Size by countries

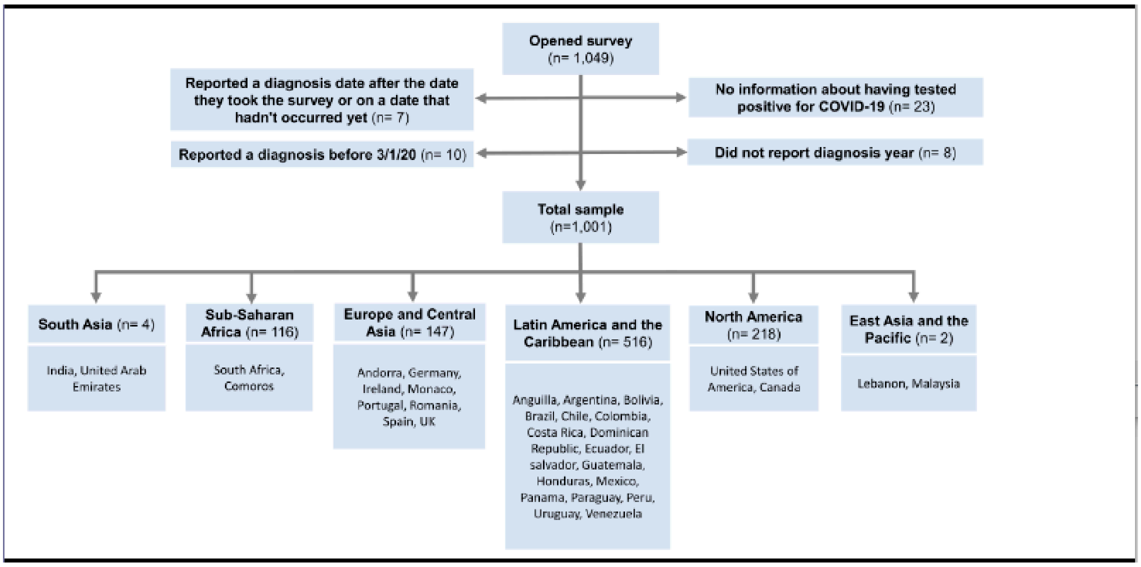

Supplementary material S2. *Bivariate correlations (*r*) between neurobehavioral symptoms and predictors.*

Variable	Somatic	Cognitive	Affective
Male Gender	-0.157**	-0.110**	-0.153**
Age	0.044	0.022	-0.010
Education	-0.123**	-0.048	-0.142**
Employed	-0.192**	-0.176**	-0.187**
Partnered	-0.064*	-0.064*	-0.051
Other Chronic Condition	0.180**	0.166**	0.208**
Hospitalized	0.254**	0.224**	0.216**
Oxygen Therapy	0.218**	0.142**	0.166**
ICU Stay	0.140**	0.069*	0.082**
Noninvasive Ventilation	0.102**	0.035	0.063*
Invasive Ventilation	0.100**	0.044	0.089**
Induced Coma	0.102**	0.050	0.100**
COVID-19 Severity	0.326**	0.306**	0.316**
Days Since Diagnosis	0.198**	0.228**	0.208**

Note. * = $p < 0.05$; ** = $p < 0.01$.
